# Service Level Agreements for 5G and Beyond: Overview, Challenges and Enablers of 5G-Healthcare Systems

**DOI:** 10.1109/access.2020.3046927

**Published:** 2021-01-05

**Authors:** HANEYA NAEEM QURESHI, MARVIN MANALASTAS, SYED MUHAMMAD ASAD ZAIDI, ALI IMRAN, MOHAMAD OMAR AL KALAA

**Affiliations:** 1 Center for Devices and Radiological Health, U.S. Food and Drug Administration, Silver Spring, MD 20993, USA; 2 School of Electrical and Computer Engineering, The University of Oklahoma-Tulsa, Tulsa, OK 74135, USA

**Keywords:** Service level agreements, 5G, healthcare, 5G-enabled medical device

## Abstract

5G and beyond networks will transform the healthcare sector by opening possibilities for novel use cases and applications. Service level agreements (SLAs) can enable 5G-enabled medical device use cases by documenting how a medical device communication requirements are met by the unique characteristics of 5G networks and the roles and responsibilities of the stakeholders involved in offering safe and effective 5G-enabled healthcare to patients. However, there are gaps in this space that should be addressed to facilitate the efficient implementation of 5G technology in healthcare. Current literature is scarce regarding SLAs for 5G and is absent regarding SLAs for 5G-enabled medical devices. This paper aims to bridge these gaps by identifying key challenges, providing insight, and describing open research questions related to SLAs in 5G and specifically 5G-healthcare systems. This is helpful to network service providers, users, and regulatory authorities in developing, managing, monitoring, and evaluating SLAs in 5G-enabled medical systems.

## I. INTRODUCTION

The key features of 5G and beyond networks, such as high multi-Gbps peak data speeds, ultra-low latency, massive device connectivity, reliability, increased network capacity, increased availability, and data-driven insights are set to revolutionize many industries and enable new applications with estimates of 1.2 billion 5G connections by 2025 [1]. One of the industries where 5G and beyond networks are expected to create a significant impact is healthcare [2]–[4].

Among the limitations of existing healthcare systems are the non-individualized diagnosis and treatment model, lack of a holistic data-driven healthcare practice model and inconvenience of transportation to access healthcare services in rural areas [5]. Additionally, medical devices commonly integrate sensors, processing logic, and actuators to be used in a single location, which limits the possibility for data reuse and efficient deployment of software updates.

Several of these challenges can be alleviated using 5G technology while creating an opportunity for augmenting current medical practices with 5G connectivity and creating novel use-cases and applications, such as telesurgery [6]–[9], accessible medical imaging, service robotics for assisted living [10], [11], in-ambulance treatment by remote physician [12], remote diagnosis/teleconsultation [13], wearable devices for different target populations such as healthy individuals, people with underlying diseases, and elderly or pediatric patients [14]. For example, 5G-enabled Internet of Things (IoT) devices might help healthy individuals in everyday routine monitoring, having a healthier life style and prevention of diseases. Patients with underlying conditions might use these devices for assisted living in chronic scenarios (e.g., glucose monitoring systems can aid diabetic patients [15]) or for follow up activities after acute events, like after a surgery. A plethora of medical device types can benefit from augmented 5G-based connectivity including vital sign monitors (e.g., electroencephalogram [EEG], electrocardiogram [ECG], electromyography [EMG], temperature, respiration, heart rate), devices using augmented and virtual reality (AR/VR), implantable devices (e.g., glucose sensor, neurostimulators), and others.

The U.S. Food and Drug Administration (FDA) guidance document on the use of radio frequency wireless technology in medical devices [16] recommends several considerations for the design, testing and use of wireless medical devices including the selection and performance of the wireless technology, wireless quality of service, wireless coexistence, and others. Compared with wireless technologies that are currently common in medical devices like Wi-Fi and Bluetooth, 5G is a centrally-managed network that expands the set of stakeholders participating to deliver the medical device functionality. Assessing and managing the risks of communication loss, delay, or disruption is complicated by the rich set of 5G features that are necessary to enable some medical device applications like network slicing where maintaining the performance of several network slices at the same time is challenging compared to the existing service assurances in legacy networks [17].

Moreover, 5G and beyond networks will operate in a multidomain, multi-operator environment with increasing number of users and varying applications with diverse requirements. Accordingly, these networks resemble an assembly of different autonomous networks, each having their own role in the service provision, their own technology and operated by separated entities [18]. Therefore, ensuring that various 5G-enabled medical devices receive the communication services needed per their unique requirements is important, especially for devices that perform critical functions (e.g., life-supporting, life-sustaining).

Documenting assurances of 5G network performance can be in the form of a service level agreement (SLA), which is a commitment between two or more parties that documents the details of various aspects of services that one party will provide to the other. This is relevant for 5G-enabled medical devices where the patient safety and medical device effectiveness depend on the 5G services provided by network operators. There are gaps in the literature regarding 5G SLAs and SLAs of 5G-enabled healthcare. Therefore, we give in this paper a brief overview of SLAs and highlight the challenges, requirements and outlook for SLAs in 5G and beyond environments and expand on SLA considerations specific to 5G-enabled medical devices.

### A. RELATED WORK

SLAs in literature are discussed in various technical domains, such as IT data centers [19]–[22], web services [23]–[27], optical communication systems [28], [29], cloud computing and IoT [30], [31].

Literature reports on SLAs for cloud computing and IoT are numerous. The studies in [32] and [33] identified over 300 existing works related to SLAs in the domain of cloud services in IoT. To present a systematic and comprehensive literature review on the topic, authors in [32] did a systematic mapping study on management of SLAs for cloud computing and IoT and categorized their findings into various SLA stages and aspects and analyzed select reports in [33]. However, the focus of [33] was not to compare the technical details of the existing literature, but to to analyze the existing literature and categorize the relevant reports with respect to their research contribution areas, maturity level of the evaluated contributions, tool support and application domains within cloud computing and IoT. Notably, the authors concluded that there are few studies focusing on concrete metrics for qualitative or quantitative assessment of quality of service (QoS) in SLAs, which highlights a need for in-depth research on metric specification and measurement methods for SLAs.

There is scarce literature addressing SLAs for 5G and beyond networks. To the best of authors’ knowledge, the only papers that discuss SLAs in this context are [17], [34]–[41].

Considering the heterogeneous nature of 5G system, the authors in [34] defined SLA parameters for 5G back-haul/fronthaul services, 5G transport network, and cloud services. These parameters include time period, periodicity, location, availability, reliability, cloud service resources, scaling rules, and operational rules. Finally, an indicative break-down of SLA monitoring functionalities is proposed based on the 5G-XHaul^1^ control plane architecture.

Authors in [35] proposed an SLA structure for 5G slice-based scenarios built on static and dynamic SLAs. Moreover, metrics of a slice-based network SLA are discussed including availability, throughput, penalty, cost, revenue and profit. The authors focused on penalty derivation, including linear versus non-linear penalty.

The advancement and optimization of the traditional SLA in a virtual environment of software defined network (SDN) were considered in [36], where the problem of mapping high-level key performance indicators (KPIs) specified by users to low-level network KPIs was addressed using data analytics and artificial neural networks (ANNs). A genetic algorithm was proposed to optimize the ANN. The authors proposed to model packet loss using the ANN as a function of bandwidth, jitter, and latency. Moreover, a mechanism for determining the importance of various Quality of Service (QoS) parameters was presented by correlating and analyzing predefined SLA template parameters, associated policies parameters and provider’s negotiation historic data. The authors claimed that the proposed mechanism might be used to improve the current negotiation, assurance and validation phases of SLAs by helping to identify dependencies between different KPIs and select the most relevant QoS metrics in the SLA. The proposed SLA management framework is part of the service platform of 5GTANGO, lead by the 5G Infrastructure Public Private Partnership (5G PPP) [42], that enables flexible programmability of 5G networks [43]. 5GTANGO consists of a service development kit (SDK) based on network function virtualization (NFV), a catalogue with validation and verification mechanisms for virtual network functions (VNFs)/network services (NSs) qualifications, and a modular service platform. For automated SLA template compilation, it is assumed in [36] that the VNF/NS are accessible in a catalogue in the 5GTANGO approach.

An extension of the work in [36] was presented in [37] and covered two additional components, namely, SLA parameter analyzer and SLA monitoring analyzer. This framework was referred to as a mediator between service providers and end-users. This work used clustering algorithms before ANNs for mapping the high-level requirements (expressed by the end users) to low-level policy (i.e., resource) parameters for the automated identification of relationships and dependencies between different parameters of datasets. A mechanism for dynamic SLA templates generation with initial SLA metrics tailored to each service provider was also proposed. Moreover, the mapping framework in [36] was extended to include complex mapping results that contain predefined formulations for the calculation of specific SLA parameters.

The assurance aspect of SLA in a 5G network slicing environment was considered in [17], where an SLA monitoring architecture based on analytical results was proposed. Correlation across different layers in a resource sharing environment was also considered. The proposed approach achieved a higher cost efficiency as compared to schemes without cross layer correlation and/or without joint monitoring analytics. However, the scheme is yet to be tested in practical scenarios and the authors described plans to test it as more practical 5G data becomes available.

Another SLA management framework utilizing 5GTANGO platform was presented in [38], [39] comprising a multi-platform web application that allows to manage the lifecycle of SLAs, on behalf of the network operator, from template creation to agreement violation detection.

Authors in [40] considered a 5G slice-aware scenario and addressed the fulfillment of SLAs. They proposed to use a mapping layer that integrates knowledge about the whole service area. This mapping layer tracks the KPIs of different slices and tunes a weighting parameter of the packet scheduler to help achieve the SLA targets for network slices. This entity is also capable of deciding slice priority. Moreover, an adaptation algorithm based on minimizing deviations from slice requirements was also proposed and the results showed improvement in the efficiency of resource sharing when the mapping layer was incorporated.

While discussing the challenges, opportunities, business and customer-centric aspects of single and multi-operator internet protocol television (IPTV) services in 5G networks, the authors in [41] highlighted the issues of lack of QoS assurance in SLAs, lack of SLA monitoring, SLA-based rewards and pricing. They proposed a framework called 5GEx, which is a wholesale service trading and exchange framework for the orchestration of network and cloud resources over multiple technological and administrative domains that aims to solve some of the issues for 5G IPTV services.

### B. CONTRIBUTIONS AND ORGANIZATION

There are gaps in literature regarding 5G-healthcare SLAs that should be addressed to facilitate the implementation of 5G-enabled medical devices. SLAs for 5G and beyond networks are addressed in a limited number of articles that primarily aim to propose specific technical solutions and the evaluation of those solutions. No previous work has comprehensively investigated whether traditional SLAs are adequate for 5G and beyond networks or detailed the challenges and limitations that can render them insufficient, which are gaps that we fill in this article. Moreover, to the best of authors’ knowledge, there is no existing work that addresses any aspect of SLAs in 5G-healthcare systems. Accordingly, the contributions and organization of this paper are detailed as follows:

- We give a brief and general description of SLAs in Section II. We begin by giving an overview of SLA definition and its importance in Sections II-A and II-B respectively. We then identify various types of current and future SLAs in Section II-C. Common building blocks of legacy SLAs are described in Section II-D. A discussion on SLA metrics, including suggestions on the types of metrics to be monitored and considerations when selecting those metrics, is presented in Section II-E. Management and monitoring of legacy SLAs are outlined in Section II-F.
- A comparison of SLAs in 5G and beyond environment with legacy SLAs is provided in Section III. Specifically, we identify why traditional SLA approaches will not suffice for 5G and beyond enabled use cases and applications and how to overcome those challenges in Section III-A. All stakeholders in 5G SLAs can benefit from this information to facilitate 5G-enabled applications, including medical devices. In Section III-B, we identify the major challenges in various stages of the SLA lifecycle in 5G and beyond networks, including challenges in the stages of SLA development, monitoring, fulfillment and assurance.
- Aspects of SLAs specific to 5G-enabled medical devices and applications are presented in Section IV. We describe the role of SLAs in medical device risk management in Section IV-A. Cybersecurity metrics and an overview of ongoing assessment and maintenance challenges is presented in Section IV-B.
- Section V concludes this paper.

## II. SERVICE LEVEL AGREEMENTS

### A. WHAT IS AN SLA?

**FIGURE 1.**
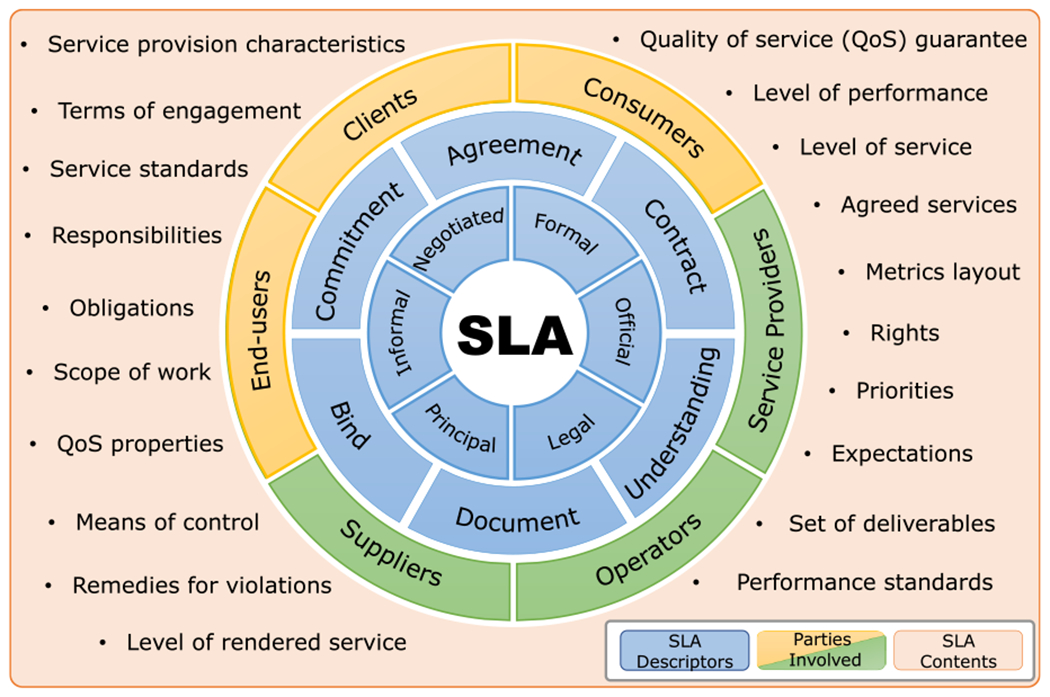
Selected SLA definitions from [35], [38], [44]–[58]. This figure should be read as follows: an SLA is [SLA descriptors (blue)] between [parties providing services (green)] and [parties receiving services (yellow)] that consists of [SLA contents (orange)].

Although sharing many similarities, relevant sources have stated various SLA definitions. Fig. 1 illustrates those definitions to highlight that at its core, an SLA is a commitment between two or more parties that documents the details of various aspects of services that one party will provide to the other. In the context of telecommunication networks, SLAs are negotiated between a consumer and a network service provider that can be an operator, an internet service provider (ISP) or an application service provider (ASP). The customer can be an ISP, an enterprise, or a subscriber (i.e., end user) [18]. For example, in the use-case of 5G-enabled telesurgery, the provider could be the network operator providing 5G services to enable telesurgery and the consumer could be the hospital that bought the telesurgery system.

### B. THE NEED FOR SLAs

An SLA protects all stakeholders in the agreement. Service providers need SLAs to help them manage customer expectations and to specify the situations under which they would not be liable for performance related issues. Customers need SLAs for assurance of guaranteed services provided to them. This provides confidence to the customer. Since the SLA describes the performance characteristics of the service, customers can also use it to compare with SLAs from competing service providers in order to select one that meets their requirements. SLA stakeholders use it as a commitment to support their interests, based on concrete, numerical goals [44]. Therefore, an SLA serves as a communication tool, a conflict resolution tool, a living document and a method for gauging service effectiveness [45], [50], [59].

### C. SLA TYPES

**FIGURE 2.**
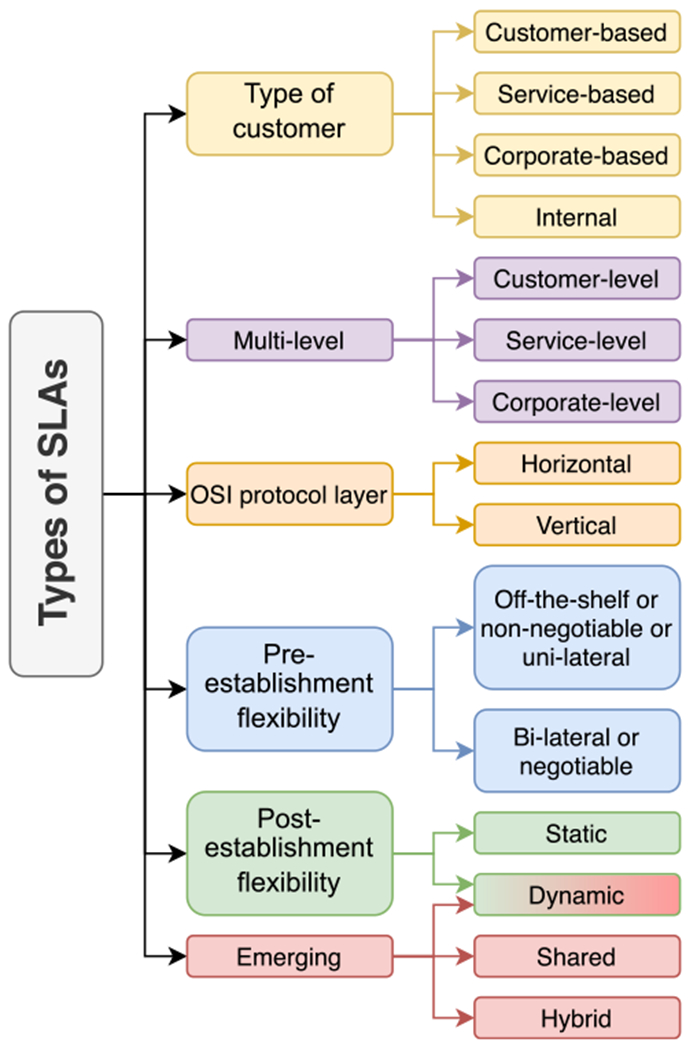
Types of service level agreements.

The types of SLAs can be organized into the groups illustrated in Fig. 2, which outlines the types that share a common theme. Considering the customer type, SLAs can be customer-based involving individual customers and covering all the services they use, service-based when offered to all customers that use the same services, corporate-based when covering all generic services for an organization, or internal when all the concerned parties are internal to a certain entity. However, a single SLA regarding a specific service could include multiple levels in the same frame (i.e., multi-level SLA) to address the service, customer, and corporate levels.

SLAs can also be categorized relevant to the Open Systems Interconnection (OSI) protocol layers. For example, a horizontal SLA can be established between peers in the same tier (e.g., SLA between two internet protocol (IP) domains or two optical transport network domains). Conversely, a vertical SLA describes the use of the underlying network layer (e.g., SLA between the core multiprotocol label switching network and an optical network) [18].

In Fig. 2, we term the SLAs that allow pre-establishment flexibility as negotiable or bi-lateral SLAs. These include negotiations during the SLA development stage. In contrast, off-the-shelf SLAs, non-negotiable SLAs, or unilateral SLAs are standard SLAs that can be commonly downloaded from the service provider’s website [60]. In this case, the customer’s role is to agree or reject the SLA. Accordingly, the lack of flexibility is not conducive to address the specific needs of mission-critical and time-sensitive applications [61].

Dynamic SLAs offer post-establishment flexibility to adapt the service level requirements and metrics in real-time. By continuously evaluating the SLA compliance at run time, the system using the service can quickly adapt to changes in its operating parameters [62]. The opposite is a static SLA, in which all components are predefined in agreement between the customer and service provider and neither party can change the service requirements for the duration of the agreement.

The last category groups emerging types of SLAs including dynamic, shared and hybrid SLAs. A shared SLA might exist in a 5G network slicing environment. The SLA is shared between a specific number of customers that use the same slice, which resembles the service-based SLA. However, the service is the network slice in this case offered by the 5G network operator. Network slicing environments in 5G and beyond networks can also benefit from hybrid SLAs, where the network slice is designed to serve certain customers first and then serve the authorized customers of the same slice [35].

### D. COMMON SLA BUILDING BLOCKS

SLAs includes components in the areas of services and management [44]–[46], [50], [56]–[59], [63]–[76]. The common components of an SLA include the agreement overview, goals and needs of involved parties, exclusions describing services that are not offered (sometimes referred to as a *force majeure* clause which aims to have zero liability on the service provider for events beyond its rational control), points of contact, supply of service, service performance measurement metrics, maintenance and repair specifications, monitoring process, service level failures and indemnification clauses [77], [78], conditions of cancellation/termination, periodic review, modifications, security and privacy management (e.g., for healthcare use cases, compliance with Health Insurance Portability and Accountability Act (HIPAA) regulations [79] is required to protect sensitive patient health information), transparency (e.g., for the medical records management or telesurgery use cases, whether the service provider will be proactive in notifying the client when the terms of the SLA are breached including infrastructure issues, like outage and performance problems as well as security incidents), certification (e.g., the customer might require that their cloud provider be ISO 27001 certified [80]), details of costs and charging methods, and finally, signatures of all stakeholders and authorized participants from involved parties to note their approval of the details and processes stated in the SLA.

### E. SLA METRICS

#### 1) WHAT METRICS SHOULD BE MONITORED?

To avoid excessive overheads to the service provider and customer, SLA metrics should be ranked according to their importance to enable a given application based on domain knowledge about the network and unique application. This is true in 5G-enabled medical devices where the importance of certain metrics might vary according to the application. For example, latency is important in a telesurgery use case. On the other hand, in a different application such as wearable IoT devices, the importance of energy consumption might outweigh latency. A simple monitoring scheme for metrics is likely to be the most effective, since the time taken for data analysis is likely to increase with complex monitoring schemes. For this reason, using automated systems for simplified collection of service metric data can reduce the cost and errors associated with manual collection of metrics.

#### 2) WHAT SHOULD BE CONSIDERED WHEN SELECTING SLAs METRICS?

The selected metrics should be SMART (i.e., specific, measurable, achievable, realistic, time-related) to avoid ambiguity [59], [65]. Measurable metrics could be developed to encourage providers and customers to adhere to the SLA terms and avoid deviations [45]. The selected metrics should also reflect factors within the service provider’s control while considering the feasibility, overhead, and cost data collection and analysis. Notably, SLA metrics that can be automatically captured and processed are less costly and generate less overhead than those requiring active monitoring and manual analysis. Other aspects that should also be considered include the data volume resulting from monitoring the selected metrics, the required resources for data analysis, metrics specification and baseline, and the likelihood that the selected metrics are sufficient to detect degradation in the established SLA terms. The challenges of monitoring performance metrics are discussed in Section III-B.

### F. MANAGEMENT OF SLAs

An SLA service level management (SLM) is responsible for ensuring that all the service management processes, operational level agreements, and underpinning contracts are appropriate for the agreed-upon service level targets [63].

In order to enforce the SLA, the specified metrics are monitored to verify whether the offered service meets the specified criteria. Three types of monitoring infrastructures are identified in [78]: 1) a trusted third-party; 2) a trusted module at the service provider; 3) a module on the client site. The monitoring mechanism should be accessible to both sides to ensure seamless service configuration, management, and maintenance [35].

Violations occur when the service level metrics in the SLA are not fulfilled. Examples and associated penalties for a resource sharing scenario in a market of computational service providers and in an optical communication system are described in [78] and [28], respectively. This topic remains open for research and contribution from technology developers and regulators, especially in high-risk 5G-medical device use-cases that are life-supporting or life-sustaining.

## III. SERVICE LEVEL AGREEMENTS FOR 5G

### A. WHY ARE TRADITIONAL SLA APPROACHES INSUFFICIENT IN 5G AND BEYOND NETWORKS?

5G and beyond networks have new and evolved technical characteristics that are not considered in existing practices of SLA generation and management. Hereafter, we describe and group these aspects based on the section of the 5G network architecture where they appear and discuss how they can be addressed in evolved 5G SLAs.

#### 1) RAN SIDE/PHY LAYER ASPECTS

5G and beyond networks are highly heterogeneous, including multi-vendor equipment, multi-operator, multi-modal environments, and multi-frequency spectrum allocations (e.g., sub-6 GHz, millimeter wave spectrum [mmWave]). Accordingly, there are new SLA considerations to the 5G radio access network (RAN) and physical layer (PHY).

Given the plethora of existing network carriers (i.e., spectrum physical resources or bearers) in the sub-6 GHz bands, the user equipments (UEs) should be camped on the optimal carrier for a given SLA service type. For example, in the case of SLAs leveraging ultra-reliable low-latency communication (URLLC), voice users should camp on larger coverage bands with commonly limited bandwidth and UEs with low latency requirements should be camped on medium bands with larger bandwidth. Accordingly, an evolved SLA should include the mechanism and guarantees for carrier association, i.e., assurance that UEs will be camped on the desired band identified for the specific use-case. In massive machine type communications (mMTC) based SLAs, searching for multiple bands can have negative implications on the energy efficiency of power-constrained IoT devices, which also can be addressed by a band selection clause in evolved 5G SLAs.

Notably, the use of mmWave spectrum contributes to enhanced 5G network capabilities compared to legacy networks. Using mmWave alleviates the capacity crunch in existing networks because of the limited spectrum available in sub-6 GHz bands. However, cell discovery in mmWave bands is challenging due to pencil-like beams, which might delay or prevent the UE from associating with a nearby large bandwidth mmWave cell. Therefore, SLAs in the 5G context, should also consider the probability of miss-association and the related impact to maintaining high download and upload speeds in SLAs leveraging enhanced mobile broadband (eMBB) use case.

Also relevant in the mmWave spectrum is the UE hand over (HO) process, especially in high mobility use cases. A successful mmWave HO completes the cell discovery process of the HO target cell including the challenging beam alignment that can be complicated by the user mobility or environmental changes like obstructions and nearby objects. Therefore, new metrics addressing cell discovery and beam alignment issues as a function of the user speed can be incorporated in evolved 5G SLAs for high-mobility scenarios.

Moreover, 3GPP specifies adaptive 5G numerology (i.e., frame structure) in order to accommodate diverse services like eMBB, mMTC, URLLC and the associated user requirements [75]. Compared to 4G networks, where the transmission time interval (TTI) is fixed to 1 ms, 5G networks can adapt the transmission by varying the TTI or symbol duration to address the desired KPI constraints, while considering the impact of UE mobility and varying channel conditions. For example, an adaptive numerology to meet the latency requirements for URLLC applications might be a subcarrier spacing of 120 kHz and slot duration (i.e., equivalent to TTI) of 0.125 ms. When TTI becomes smaller, the signals will be transmitted in a larger bandwidth since frequency is inversely proportional to time scale. Due to larger signal bandwidth, the channel will be more susceptible to frequency selective fading, which occurs when the signal bandwidth becomes larger than the coherence bandwidth of the channel. A consequence of frequency selective fading is that different frequency components in the signal get attenuated by different amounts, which limits the range of communication or cell radius. Therefore, larger TTI is suited for eMBB/mMTC use cases or use cases that require a larger radius, but with smaller TTI, lower latency can be achieved at the cost of reduced cell size. Another factor to consider is the subcarrier spacing where a small value leads to a short TTI, which might be desirable for quick transmissions and hybrid automatic repeat request (HARQ) feedback. Hence, in contrast to legacy SLAs, SLAs for 5G and beyond should consider the TTI constraints to ensure the harmony between the application requirements and network capabilities (e.g., a conflict arising when the SLA specifies 0.125 ms TTI but the network is configured to support 1 ms TTI).

**FIGURE 3.**
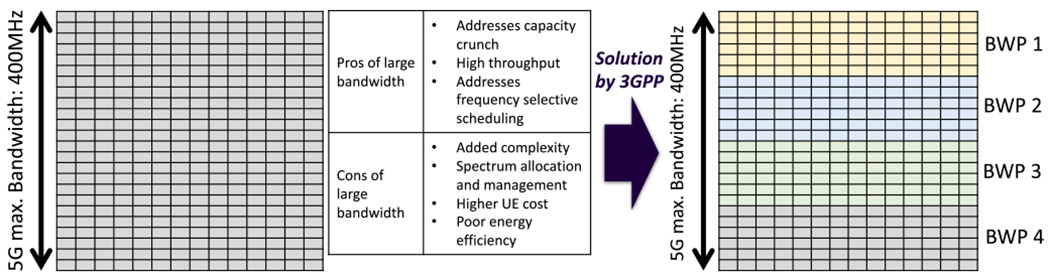
The concept of 5G bandwidth adaptation.

Another 5G physical (PHY) layer aspect is the division of spectrum into the bandwidth parts specified in 5G new radio (NR) as illustrated in Fig. 3. A static bandwidth allocation close to the upper end of possible values (i.e., 400 MHz) is challenging for IoT devices and sensors having low power and low processing capabilities that are typical in mMTC applications. Therefore, the introduction of bandwidth adaptation in 5G can provide flexibility and facilitate power saving. This highlights the importance of considering energy efficiency in 5G SLAs and how it relates to the bandwidth allocated to the user by the 5G network to ensure a desired application receives adequate network resources and avoid being under-scheduled.

**FIGURE 4.**
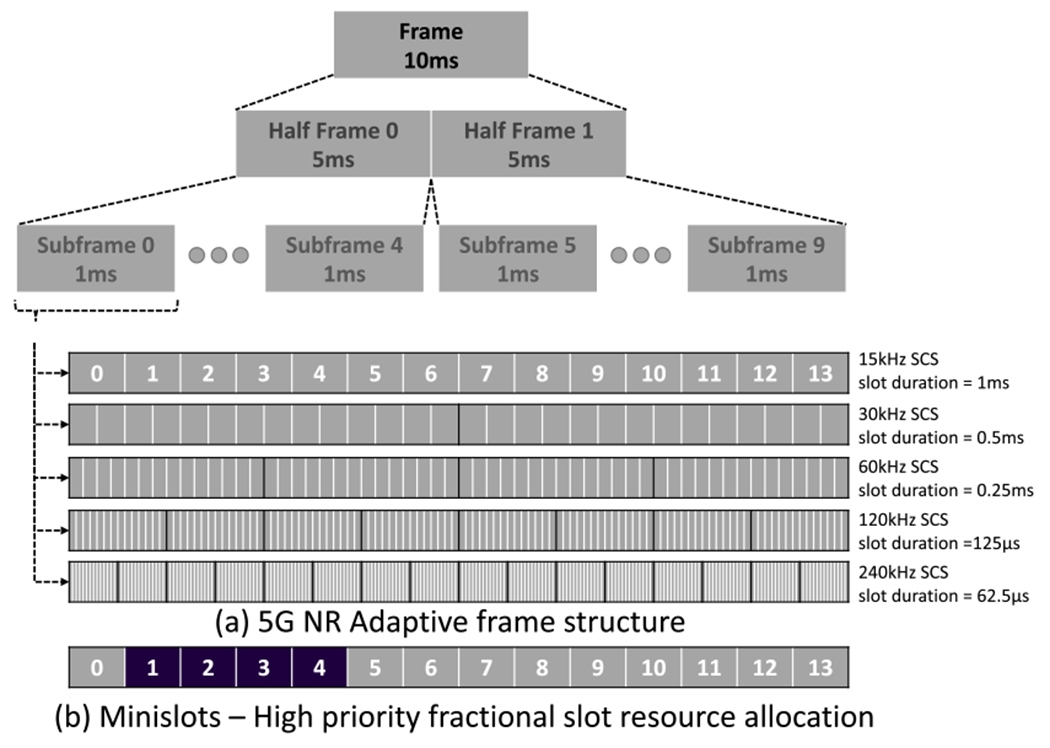
5G adaptive numerology and minislots.

4G LTE networks perform resource allocation as multiples of one time slot, where 1 slot = 1 ms = 14 orthogonal frequency-division multiplexing (OFDM) symbols. 5G introduces the concept of mini slots where a UE can be allocated resources on the symbol level (e.g., 2, 4 or 7 symbols in a minislot). The concepts of minislots and adaptive numerology are illustrated in Fig. 4. Also possible in 5G is aggregating slots to reduce the signaling overhead during resource allocation. Instead of acknowledging every physical resource block (PRB) separately, ACK/NACK are sent for a group of PRBs due to slot aggregation. Moreover, minislots can pre-empt normal transmissions, which can be useful for URLLC services and time-critical communication. Accordingly, 5G SLAs can be augmented to consider limits on the variable allocated resources, i.e., how many symbols in a mini slot are needed and would be provisioned for a specific service, whether slot aggregation is allowed, and whether and how frequently minislot pre-emption is allowed.

#### 2) CORE SIDE/NETWORK LAYER ASPECTS

5G network slicing is an innovative flexibility in the network architecture to facilitate the provision of 5G network resources according to specific SLAs. Network slicing permits the partitioning of network architecture into virtual elements, such that each virtual element is suited for a specific use-case or SLA. However, to enable SLA assurance and verification, the network performance data collected to establish SLA KPIs should address the network slice which can be different from the data collected for the overall network.

Unlike SLAs in legacy telecommunication networks that share many similarities resulting in similar SLA metrics, slice-based 5G networks can offer unique services that can be addressed in a per-slice SLA approach, where individual SLAs have unique elements, metrics and structure. Notably, the business model, SLA structure, QoS specifications, cost model, and the level of service can differ between slices [35]. Accordingly, new scheduling and resource allocation mechanisms (e.g., via weighted slice distribution strategy) and network admission control policies can be considered in the per-slice SLA. Other types of SLAs that can be applicable in a 5G network slicing environment include shared SLAs (i.e., shared between specific number of customers that use the same slice) and hybrid SLAs (i.e., expected to serve certain customers first and then serve the authorized customers of the same slice [35]).

5G and beyond networks are dynamic and can adapt the provided service according to the customer demand for specific KPIs. Accordingly, dynamic SLAs should be considered to capture the limits within which the service provider and customer will operate. An example of dynamic service provisions is those of cloud services where the provider offers cloud facilities in various modes that are capable of scaling up or down in real time to meet the customer demand for resources. This flexibility is coupled with a dynamic change in the SLA QoS parameters [81]. Another example is a telesurgery platform requiring low-latency communication for the duration of the procedure, i.e., the customer can be charged for a network slice to meet their demand for latency and bandwidth for the duration of the surgery. However, when the surgery is complete, the customer would invoke the mechanism specified in the dynamic SLA with the service provider to change their demand for network resources [35].

### B. CHALLENGES IN DIFFERENT STAGES OF SLAs IN 5G ENVIRONMENT

**FIGURE 5.**
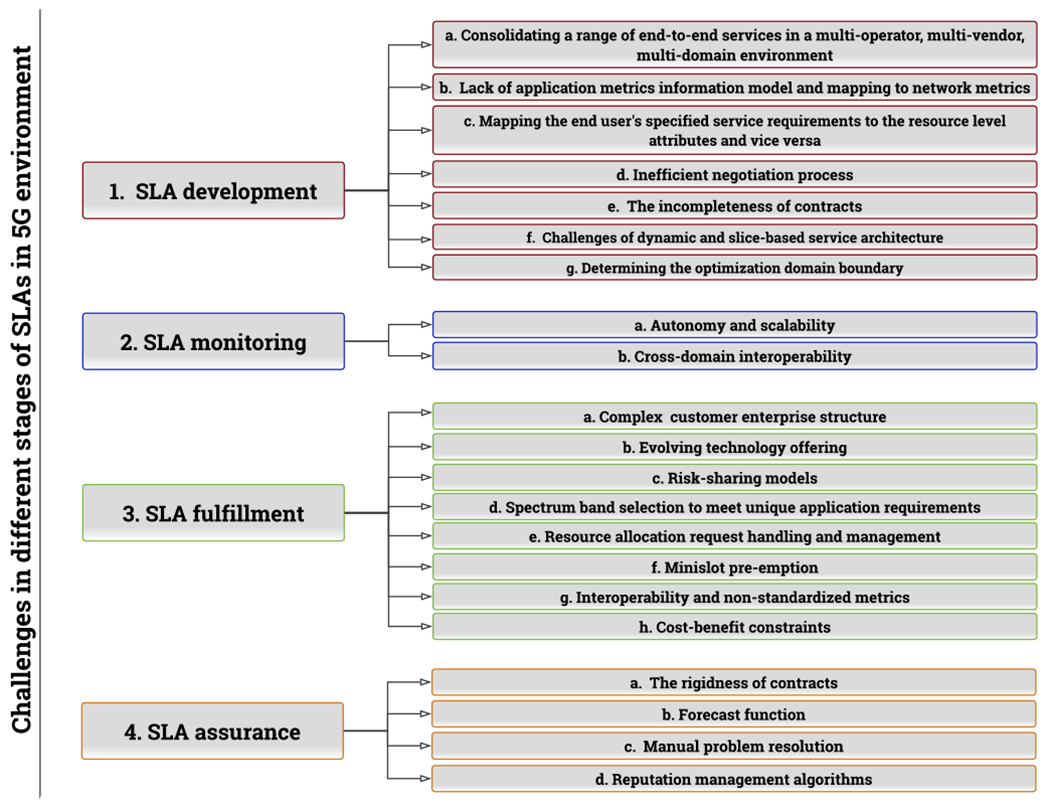
Challenges of 5G SLAs categorized according to the development, monitoring, fulfillment, and assurance SLA parts.

Evolved SLAs for dynamic 5G and beyond networks are more complex than existing ones in terms of agile network management to accommodate novel applications and dynamic QoS requirements. In this section, we identify and describe 5G SLA challenges during the various stages of the SLA lifecycle. These challenges are illustrated in Fig. 5 and are described below. Notably, there is a correlation between the challenges identified in this section given the common theme of dynamic and heterogeneous 5G and beyond networks.

#### 1) CHALLENGES IN SLA DEVELOPMENT

Specifying the customer communication needs and mapping those needs to the 5G network technical capabilities establish the theme of challenges during the SLA development that include the following:

##### a: CONSOLIDATING A RANGE OF END-TO-END SERVICES IN A MULTI-OPERATOR, MULTI-VENDOR, MULTI-DOMAIN ENVIRONMENT

In 5G and beyond networks, the service is provided as a result of a multi-stakeholder collaboration that involve multiple network technologies. Ownership of the entire ecosystem is commonly not held by a single entity. Outsourcing of service functions is expected to increase in the 5G business model to save costs, reduce risk, or to benefit from specialized service providers [82]. In this case, networks providers lease parts of their networks, which can be managed through agreements with the lessees and between providers and end customers. Accordingly, delivering a desired service to the end customer involves processes for alignment and coordination between the various involved service providers. This highlights the opportunity to establish methods for developing SLAs where multiple parties are involved in the service delivery. One work in this direction is proposed in [83]. Other propositions in this context are given in [18], where two scenarios are identified to provide an end-to-end service for an end-user: (i) the end-user must manage different SLAs and is the only one who manages their interactions from end-to-end; (ii) the end-user manages only one SLA with a service provider and all necessary information for service management is propagated into the network from end to end, including out-sourced components. Furthermore, it is not straightforward to implement an end-to-end service level management system that can accurately and granularly measure network performance in a 5G environment with varying logical architectures, functional splits, and QoS needs across network layers [18].

##### b: LACK OF APPLICATION METRICS INFORMATION MODEL AND MAPPING TO NETWORK METRICS

Considering the application side, information models or templates might not exist to identify the communication performance metrics and other technical details that are needed to fulfill the intended functionality of the plethora of 5G service types and applications. Such templates help the stakeholders to cooperate and negotiate tradeoffs to facilitate service delivery. On the network side, choosing a configuration of network parameters to meet the desired application performance can benefit from a mapping between the SLA metrics and 5G network parameters that highlights the sensitivity of desired performance to the change in network configuration. This can be accomplished by leveraging domain knowledge in both the service application area and 5G network management, which exceeds in complexity compared to the legacy networks because of the increase in the number of network parameters and their complex interdependencies. The work in [18] attempts to map ten services to ten network technology independent parameters by considering four performance classes: 1) very high performance, 2) high performance, 3) default performance, and 4) indifferent. However, this work does not consider 5G applications and metrics.

##### c: MAPPING THE END-USER’s SPECIFIED SERVICE REQUIREMENTS TO THE RESOURCE LEVEL ATTRIBUTES AND VICE VERSA

The exchange of information between SLA stakeholders becomes challenging with the increase in number and business interests of the stakeholders. Accordingly, reaching a compromise that satisfies the SLA requirements can benefit from a precise mapping of the customer high-level communication requirements (e.g., achieving a specific latency value for a telesurgery platform) to the low-level network KPIs and network policy resource-level attributes [36]. This helps bridge the gap between the expectations of customers and service providers and facilitate negotiation clarity between stakeholders in the SLA development phase. The studies in [37] and [36] aim to address this challenge using data analytics and artificial neural networks to automatically identify the interdependencies between different parameters. A framework that implements the reverse process is proposed in [84], where the authors address the translation of low-level metrics to high-level SLA terms that are used in cloud service level agreements.

##### d: INEFFICIENT NEGOTIATION PROCESS

Manual negotiations of SLA metrics and service assurances can be inefficient. This is especially true in 5G and beyond networks due to the increased complexities highlighted earlier in this section. Accordingly, it is likely that automated inter-domain negotiation processes will be developed and used to determine the importance of different KPIs by analyzing the predefined service parameters while leveraging historic data documenting the service provider’s negotiations [36]. This approach also helps focus on the most relevant KPIs for a certain application for inclusion in the SLA.

##### e: THE INCOMPLETENESS OF CONTRACTS

SLAs are inherently limited by the technical scenarios envisioned upon SLA creation. Hence, changing requirements might lead to situations that are not covered by the SLA terms. Furthermore, verifiable data can be challenging to obtain for service level specification. Accordingly, it is not uncommon to find qualitative statements such as “as soon as possible” in the SLA [85]. These gaps in SLA coverage could result in conflict, which highlights the importance of transparency, ongoing communication, and cooperation between the SLA stakeholders.

##### f: CHALLENGES OF DYNAMIC AND SLICE-BASED SERVICE ARCHITECTURE

While network slicing contributes to maintaining cost-effective network operations, it is challenging for the network operator to allocate portions of the network on-demand. The trade-off between static and dynamic network slicing, which is also applicable to static and dynamic SLAs, involves network efficiency, complexity, and cost. In a static slicing scenario, simplicity is achieved by configuring the network once to allow users continuous access to the allocated network resources without impacting other slices. However, cost and network efficiency are sub-optimal considering that users allocated to a busy slice cannot benefit from the resources available in an idle slice. Dynamic network slicing on-demand can alleviate this inefficiency. However, the challenge is to decide when and which slices to pre-empt to provide the users in the slice covered by the SLA with the agreed services. Moreover, accurate SLA assurance verification in a slice-based environment relies on per-slice KPI monitoring, which should be clearly captured in the SLA.

##### g: DETERMINING THE OPTIMIZATION DOMAIN BOUNDARY

The SLA stakeholders should consider the limits of their influence on the network optimization strategies and the impact of those strategies on the services promised to the customer and the services provided by the network operators to other customers.

#### 2) CHALLENGES IN SLA MONITORING

Revolving around the task of capturing useful data streams in a heterogenous network to facilitate adequate SLA monitoring, we describe the following challenges of SLA monitoring in 5G and beyond environments.

##### a: AUTONOMY AND SCALABILITY

Manual monitoring of SLA parameters can be expensive, time-consuming, and unscalable. Although the automated monitoring tools used by network operators could be leveraged to support SLA monitoring, access to these tools is commonly reserved to the internal use of the service provider. Using common signaling (e.g., generalized multi-protocol label switching) with a generic policy manager or a third party can help automate the SLA monitoring tasks. However, this will include the added burden of mapping the SLA requirements of each SLA to the technical configurations of network equipment used by the service provider and the specification of tools to generate SLA performance metrics [18].

Another challenge is the data volume resulting from data collection for SLA monitoring. Service quality metrics are specified based on detailed infrastructure-based measurements that can generate large volumes of data, which is challenging for customers to analyze and determine the service consistency with the SLA terms. To alleviate the burden of analyzing large data volumes, the stakeholders can identify the most important and relevant data stream and only gather the associated technical reports for assessment. Although this approach can reduce the administrative burden on the SLA stakeholders, there can be cases where the customer requires detailed data collection for traceability and compliance with external reporting commitments. The importance of SLA monitoring automation is further highlighted by the large number of technical counters in heterogenous 5G and beyond networks, the use of vendor-specific monitoring tools by network operators, and the lack of unified data format for collected data. Accordingly, a gap in the existing methods is the lack of automated SLA monitoring methods that are capable of efficiently addressing the SLA monitoring tasks of 5G and beyond networks. Automated, scalable, and transparent data collection and aggregation helps build trust between SLA stakeholders and promotes efficient use of resources to achieve the customer desired application.

##### b: CROSS-DOMAIN INTEROPERABILITY

SLA monitoring methods for 5G and beyond networks should account for cross-domain monitoring involving multiple organizations (e.g., network operators, connectivity outsourcing companies), systems, and entities (e.g., network performance monitor, service and application monitor, virtualization manager or storage manager). Therefore, methods should be considered to permit management information flow across administrative domain boundaries and facilitate an end-to-end view of the service provision in a common platform that promotes cooperation between multiple organizations and integrates multiple domain monitoring modules. However, the lack of standardized performance metrics for use in data collection and aggregation hinders the automation and interoperability of such platform across multiple domains for 5G SLA monitoring.

#### 3) CHALLENGES IN SLA FULFILLMENT

SLA fulfillment is closely related to SLA monitoring. However, the impact of business needs and expectations of the SLA stakeholders highlight the challenges listed below.

##### a: COMPLEX CUSTOMER ENTERPRISE STRUCTURE

In complex company structures, it is challenging to correlate the quality of services in terms of business value creation. With growing enterprise complexity, the number of internal customer entities increases along with their inter-dependencies and potentially conflicting requirements. When a value model for the procured services is absent, the sensitivity of the business value of a desired application to service changes is not easily predictable.

##### b: EVOLVING TECHNOLOGY OFFERING

Customers might attempt to improve their connected applications to remain competitive (e.g., serve more subscribers, increase access speed to medical imaging data). However, there is no financial incentive for the service providers to offer technical capabilities beyond what is needed to meet the established SLA terms. Accordingly, evolving the technology offered by the service providers can be regarded as a challenge since such investment in service quality can impact the provider’s cost structures. Customers wishing to expand their access to improved technology should initiate a new negotiation process with the service provider [85]. Therefore, the customer should maintain up-to-date technology landscaping efforts in the evolving 5G and beyond networks to be aware of what can be done with improved communication capabilities. On the other hand, the service providers can benefit from the targeted marketing of their communication service offerings to industry verticals.

##### c: RISK-SHARING MODELS

Business costs and success can be perceived differently by the SLA stakeholders, which extends to the associated risk to that success. Accordingly, the SLA stakeholders should determine if and how to consider risk-sharing of the end-to-end service provided to the end-user. Unique industry verticals can approach this topic according to their unique needs. We expand on the risk management of 5G-enabled medical devices in Section IV.

##### d: SPECTRUM BAND SELECTION TO MEET UNIQUE APPLICATION REQUIREMENTS

Due to an increasing number of sub-6 GHz carriers in 5G and beyond networks, a challenge for service providers is to ensure that users are camped on the optimal carrier in 5G according to the service type. Spectrum bands in 5G networks are divided into low, medium, and high bands corresponding to less than 1 GHz, 1 GHz to 6 GHz, and 24 GHz to 40 GHz, respectively. Band selection is important because it ensures minimum inter-frequency hand overs by avoiding measurement gaps, which is the key contributor to voice muting occasions (i.e., due to cell radio shifting to another carrier during measurement gaps). In 5G voice services, the biggest problem is call muting, rather than call dropping or call quality. Muting is a gap in voice packets or real-time transport protocol (RTP) packets, which is perceived by human ears as silence. Call dropping means that a call ends unexpectedly. However, in 5G packet-based voice service, with VoLTE, users are left on the receiving end of silence (i.e., go mute during the call) due to loss of voice packets. Packet loss has a pronounced impact on time-critical applications with low bandwidth requirements whose users would expect to be camped on a low spectrum band with relatively small bandwidth. However, low bands are congested with 2G, 3G, 4G and other services. Accordingly, medium-band with larger bandwidths compared to low-band can be considered for time-critical applications (i.e., SLAs for URLLC use cases).

##### e: RESOURCE ALLOCATION REQUEST HANDLING AND MANAGEMENT

In 5G and beyond use cases, provisions like spectrum sharing and infrastructure sharing complicate the resource allocation in SLA management. For example, short-term services requested through signaling can be challenging to meet by the service provider because of the complexity of managing the network resource reservation while balancing the overall services offered to all customers and maximizing resource utilization [36]. Bandwidth adaptation in 5G and beyond networks and how it can impact the desired application should also be considered and documented in the SLA.

Managing spectrum sharing scenarios would be a challenge as well. If used, spectrum sharing practices should be addressed in the SLA, whereby some service providers might consider the temporary transfer of some or all their spectrum access rights. Furthermore, the optional use of unlicensed spectrum bands is commonly best-effort and lacks service guarantees due to the lack of interference protection in unlicensed spectrum, which raises concerns for wireless coexistence. For example, the coexistence impact of LTE-Licensed Assisted Access (LAA) on users of unlicensed spectrum including wireless medical devices was investigated in [86]. Authors in [87] address the problem of modeling and evaluating the coexistence of LTE LAA in the unlicensed band. Accordingly, considerations of wireless coexistence should be addressed in the SLA if applicable to the offered service.

Another SLA consideration is the network physical resource sharing and its impact on the offered service. Often, a customer does not need a high QoS at all times. For example, in the case of connected ambulance facilitating patient treatment by a remote physician while in transport, the service level needed to operate the associated connectivity would only be needed while the patient is on the way to hospital.

Once the patient reaches the hospital, that communication service is no longer needed. For such applications, customers can request on-demand services that are charged on a pay-as-you-use basis, which might be an incentive for the provider to share the network resources between users to achieve profitability [85].

##### f: MINISLOT PRE-EMPTION

In URLLC use-cases, 5G minislots can pre-empt normal transmissions, which can be useful when there is a need for time-critical communication. However, pre-emption can negatively impact other network users, e.g., a user will be affected if its transmission is pre-empted because of another higher priority user. Therefore, the SLA should consider the trade-offs of using minislot pre-emption that are application specific and lack established best practices.

##### g: INTEROPERABILITY AND NON-STANDARDIZED METRICS

Interoperability should be considered between the various components of the 5G-enabled medical device application [88] in addition to the interoperability between various network equipment vendors to facilitate SLA service delivery. Interoperability challenges for SLA fulfillment are further highlighted by the fact that network performance metrics are commonly vendor-specific, where each network equipment vendor defines metrics using its own set of counters and naming conventions. In addition to managing non-standardized network performance metrics, SLA fulfillment includes the challenge of translating the customer requirements to technical specifications [85], which can be presented as customer business goals. In this case, the SLA stakeholders develop a mapping between the technical and business metrics to align the SLA with their business goals and document the expected business value contribution of the measurable network performance metrics. Business metrics indicate the progress of a stakeholder’s goals and can include metrics for marketing (e.g., incremental sales), sales (e.g., average profit margin), financial value (e.g., debt-to-equity ratio), software as a service (SaaS, e.g., customer retention rate), or social media (e.g., number of twitter followers) [89], [90].

##### h: COST-BENEFIT CONSTRAINTS

The customer budget might limit the level of service obtained from the network service provider. Accordingly, the challenge is to maintain a tolerable customer cost-benefit ratio including the cost assessment of possible technical solutions that can meet the customer expectations and the associated trade-offs.

#### 4) CHALLENGES IN SLA ASSURANCE

This part of SLA management assures that the provided service achieves the performance set in the SLA.

##### a: THE RIGIDNESS OF CONTRACTS

While foreseeable future requirements are considered during SLA development, the unpredictable change in customer requirements is challenging to address for SLA assurance. Unpredictable requirements encountered during the lifecycle of SLAs complicate the SLA applicability to evolving customer needs where the established correlations might become outdated between business needs, network performance metrics, and cost. Notably, the incentive to adapt an SLA to new situations decreases as the contract period nears its end [85]. Accordingly, considering dynamic SLAs in 5G and beyond networks can help prepare the stakeholders to address evolving technical and business situations during for the SLA duration.

##### b: FORECAST FUNCTION

An open research question is the development of continuous network forecasting and optimization techniques to optimize a set of desired network aspects (e.g., coverage, energy efficiency, spectral efficiency) based on variable inputs (e.g., traffic, environmental factors). Although there are reports on advancements in this area, it is unclear what the optimal mapping is between the proposed forecasting techniques and network parameters [18]. However, in dynamic 5G and beyond networks, forecast functions are central to the deployment of features like network slicing, where the network resources are dynamically optimized between slices to improve utilization while meeting the SLA service levels [37]. Hence, the challenge is to develop, deploy, and document a forecast function that meets the optimization objectives and constraints for every network slice with the available input streams.

##### c: MANUAL PROBLEM RESOLUTION

With increasing complexity and heterogeneity of 5G and beyond networks, the lack of automatic problem resolution is challenging. To facilitate efficient service problem resolution, automated tools can be useful in root cause analysis, trouble ticketing, and traffic forecasting.

##### d: REPUTATION MANAGEMENT ALGORITHMS

SLA penalties can negatively impact the service provider reputation [91]. This is augmented in cases where the service performance metrics include client reviews. In 5G and beyond networks, a challenge in the review-based evaluation can be the ease of generation of large volumes of dummy clients by a service provider to build their reputation or damage the competition. For example, due to posting fraudulent reviews against its competitor HTC, Samsung was fined $340,000 in 2013 by the Taiwan Federal Trade Commission [92]. The interested reader is referred to the comprehensive study in [93] for more information on this topic.

## IV. SERVICE LEVEL AGREEMENTS FOR 5G ENABLED MEDICAL DEVICES AND APPLICATIONS

### A. RISK MANAGEMENT

Risk management is a key component of medical device design including 5G-enabled medical devices and healthcare systems. Accordingly, it is important to consider the risks associated with 5G communication loss, delay, or disruption that might lead to a hazardous situation.

A risk management process is specified in ISO 14971 standard for application of risk management to medical devices [94]. In summary, throughout the medical device life cycle, the process includes the identification of hazards, evaluation of associated risks, risk controls, and monitoring the risk controls effectiveness. In addition to supporting device safety, comprehensive risk management helps device developers optimize efficiency and reduce costs [95]. 5G-enabled medical devices are emerging at the intersection between the medical device and telecommunication industries, which highlights the increasing technology convergence in modern society and illustrates the benefit of crossing the boundaries of existing knowledge and practices between industry domains. Hence, we note the similarities between the ISO 14971 risk management process and SLAs as described in Section II. Accordingly, an SLA implemented to facilitate a 5G-enabled medical device is a part of the overall medical device risk management process that is specific to its 5G use. The SLA includes the identification of needed level of service that corresponds to safely achieving a desired medical device function and the mitigation strategies to control degraded communication service. It can also inform the risk management process through the specification of quantitative metrics and the relationship between network performance metrics and medical device function. For example, the requested service time for a given application might be required below a threshold to maintain an acceptable risk. Therefore, the SLA addresses the desired metric by highlighting its dependence on the mean-time-to-failure [96] of the underlying network services and stating the assurances the network provider implements to guarantee QoS.

#### B. CYBERSECURITY

Connectivity is widely used in healthcare system with estimates that 74% of total hospital equipment are connected medical devices [97], which highlights the concerns for medical device cybersecurity. In this regard, the FDA published a draft guidance on the content of premarket submissions for management of cybersecurity in medical devices [98], which provides recommendations to industry regarding cybersecurity device design, labeling, and the documentation that FDA recommends be included in premarket submissions for devices with cybersecurity risk. The FDA draft guidance addresses aspects of medical device cybersecurity management, such as risk assessment, designing a trustworthy device (e.g., identifying and protecting device assets and functionality), device labeling recommendations, and cybersecurity documentation. In this section, we focus on the cybersecurity aspects of the 5G technology that enables 5G healthcare applications.

The network architecture of cellular systems present unique cybersecurity challenges compared to short-range wireless technologies like Wi-Fi, ZigBee or Bluetooth Low Energy [99], [100]. Although cellular networks embed security processes, they are not immune to malicious attacks like eavesdropping and message spamming in the analog first generation networks and 2G systems. IP-based network like 3G and 4G suffer from a variety of attacks including cryptographic attacks, denial of service (DoS), network impersonation, man-in-the-middle (MITM), and spoofing [101]. These and new types of attacks can be expected in 5G where the technical advancements enabling 5G capabilities form new attack vectors. For example, intrusion detection can become challenging with the large number of heterogenous equipment connected to a 5G network. High connection throughput could allow attackers to quickly download big volumes of data (e.g., patient information and medical imaging data) in a compromised network. Low latency 5G connectivity facilitated by mobile edge computing infrastructure can enable medical device applications like telesurgery, where a DoS attack can result in patient harm.

Based on the reports presented in [102]–[105], we compiled a list of potential cybersecurity threats in 5G. The threats are categorized based on the susceptible 5G system component. Table 1 shows the types of threats in 5G networks, the point of attack, and the affected healthcare application if the attacks are successful. The reader can refer to [106] for a detailed discussion of the 5G network architecture. Notably, medical IoT devices are susceptible to the attacks on the UE and RAN side of the 5G network. Remote medical procedures use 5G low latency capabilities, which are enabled by edge computing. Therefore, they are susceptible to attacks on the 5G cloud edge. Given the variety of potential threats and attack modalities, we discuss hereafter cybersecurity metrics and ongoing assessment and maintenance in 5G SLAs for connected healthcare systems.

##### 1) METRICS

Cybersecurity metrics contribute to building the 5G network reliability and trustworthiness [99], [107]–[109]. Any network, wired or wireless used to transfer information should provide some degree of safety. In case of 5G network catering healthcare-related traffic and data of great significance, this aspect becomes essential. Examples relevant for consideration in SLAs of 5G-enabled medical devices (some of which are also very important in 4G applications) include:

1. Authenticity: the 5G network should establish the authenticity of devices or users requesting access and determine whether they are legitimate or cyber adversaries.
2. Confidentiality: limiting the access of unauthorized users to data whether passing through the 5G network or stored in the cloud.
3. Integrity: preserving the data accuracy and reliability and preventing falsifications and unauthorized modifications. Continuous monitoring helps ensure data integrity and detect adverse events.
4. Availability: closely related to confidentiality, availability corresponds to the ability of the system to provide data to its legitimate users whenever and wherever requested. Availability degrades when a legitimate user is trying to access the 5G wireless network but is unable to do so because of cyber adversaries.
5. Vulnerability: refers to the potential for cybersecurity breach, i.e., a measure of the 5G network weakness to malicious cybersecurity attacks.
6. Agility: a measure of the network ability to adapt to evolving cybersecurity needs and implement up-to-date response strategies.
7. Resilience: a measure of the network ability to mitigate cybersecurity attacks within a given time to avoid service disruption and data corruption.
8. Mean time to detect: a measure of the time needed by the cybersecurity system to detect a potential security breach.
9. Mitigation/recovery time: a measure of the time needed by the cybersecurity system to mitigate an attack or security breach, eliminate further risk, and return to normal operation status.
10. Proactiveness: a measure of the cybersecurity system ability to foresee a potential threat before it occurs and implement proactive mitigation actions.

##### 2) ONGOING ASSESSMENT AND MAINTENANCE

As an extension to the stakeholders’ roles identified in the SLA, cybersecurity aspects can be considered to identify those responsible for the ongoing threat assessment of the 5G service enabling a healthcare application, mitigation strategies, and implementing response actions when applicable. Given the wide range of possible applications, overseeing the overall system cybersecurity can be shared between the SLA stakeholders including the end-users (e.g., patients, hospitals, healthcare professionals), network service providers, and third parties tasked with network security assurance. Addressing cybersecurity in the SLA promotes transparency, facilitates communication between the involved stakeholders, including their internal entities (e.g., teams for network planning and network monitoring), establishes consensus practices to solidify the overall system security, and expands the scope of considerations that a customer can review when planning to procure the 5G service.

The SLA terms for security management and maintenance can include plans to mitigate known threats (e.g., mitigating DoS attacks by using redundant communication links for the offered service) and respond to future threats (e.g., upgrading network firewalls to limit the potential for data theft, considerations to push remote updates to the medical device software and firmware). Innovative methods can be considered, where applicable, for encryption key management (e.g., electrocardiography-based key generation), probabilistic framework for risk assessment [110], and using artificial intelligence for anomaly detection to prevent service disruption, eavesdropping, and signal jamming [111]. The impact of the SLA cybersecurity terms on the healthcare application should also be considered. Examples include the device resource constraints (e.g., energy consumption and computing power) to meet given network access requirements (e.g., secret key storage and signaling).

## V. CONCLUSION

5G and beyond networks will transform the healthcare industry by enabling novel use cases and applications, such as telesurgery, remote patient diagnosis, smart medication, and healthcare big data management, and promote new medical device modalities where 5G capabilities enable the integration of distributed device sensors, actuators, and processors. Facilitating patient access to novel 5G-enabled medical device applications requires that devices integrate 5G technology safely and effectively to deliver the intended device function. As a part of the overall medical device risk management process, SLAs are a framework for documenting the communication requirements for diverse 5G-healthcare use-cases and specifying the roles of all stakeholders to ensure that the delivered 5G service meets the customer expectations. In this article, we present an overview of SLAs, identify the challenges for SLAs in 5G and beyond networks, highlight practical aspects for SLA development and implementation, and recommend considerations to help enable 5G-healthcare systems.

Developing an SLA for a given 5G-enabled medical device offers all stakeholders the opportunity to consider the risks associated with the communication service degradation, delay, or disruption and what risk mitigation strategies can be implemented on the network side to help control those risks. Although the underlying biomedical and communication technologies have seen significant advancements leading to their convergence in 5G-enabled healthcare, we identified open questions that the research community can help answer to promote the safe use of 5G and beyond communication technology in healthcare. These include topics, tradeoffs, and practical implementation considerations in 5G network resource allocation like provisioning minislots for a specific service, optimal triggering of minislots pre-emption, optimizing device performance when using bandwidth adaptation, network slice sharing modes, and dynamic network resource optimization. Research is also needed to understand the integration of UE miss-association probability to mmWave cells in the medical device risk evaluation and strategies to address it in the SLA. With increasing network complexity, the need arises for adaptive algorithms to reduce the large set of observable network counters and metrics and facilitate efficient network monitoring for service assurance. Additionally, algorithms are also needed to flexibly map and optimize network configuration parameters to meet desired healthcare application while maintaining business objectives for all stakeholders. This can also extend to facilitate dynamic SLA negotiation and implementation practices for evolving customer needs. The heterogenous and multi-domain nature of 5G and beyond network illustrate the opportunity to develop collaboration frameworks to promote interoperability and service delivery. In addition to their applicability in other industry verticals, addressing those research challenges promotes the safe integration of 5G and beyond technology in healthcare and the development of robust SLAs to ensure that device manufacturers, network service providers, and regulators share a common framework for healthcare service delivery.

## Figures and Tables

**TABLE 1.** Cybersecurity threats to the 5G ecosystem with the corresponding affected healthcare applications.

Point of attack	5G security threats and attacks type [102]–[105]	Affected Healthcare Application		
		Medical IoT (e.g., wearables, implantable devices, on-site equipment, home-based medical devices)	Remote Medical Procedures (e.g., telesurgery, teleconsultation, ambulance drone, telemedicine, in-ambulance treatment)	Medical Data Management (e.g., confidential health records, personally identifiable information)
Internet/Other Operator	Security Policy Conflicts		**x**	**x**
Internet	Pharming		**x**	**x**
	Trojan		**x**	**x**
	Virus		**x**	**x**
5G Central Cloud	Hijacking Attack		**x**	**x**
	Man-in-the-middle Attack		**x**	**x**
	Configuration Attack		**x**	**x**
	Saturation Attack		**x**	**x**
	Signaling Attack		**x**	**x**
5G Central Cloud/5G Edge Cloud	Slice/Resource Theft		**x**	**x**
5G Central Cloud/5G Edge Cloud/5G RAN	Distributed Denial of Service (DDoS) Attack	**x**	**x**	**x**
	Denial of Service (DoS) Attack	**x**	**x**	**x**
	Penetration Attack	**x**	**x**	**x**
5G RAN	Reset and IP Spoofing	**x**	**x**	
	Scanning Attack	**x**	**x**	
	Semantic Information Attack	**x**	**x**	
	Signaling Storms/Signal Jamming	**x**	**x**	
	International Mobile Subscriber Identity (IMSI) Catching Attack	**x**	**x**	
	Illegal Intercept	**x**	**x**	
	Flash Traffic	**x**	**x**	
	Fake Base Station Attack	**x**	**x**	
End user	User Identity Theft	**x**		
	Security Key Theft	**x**		
	Advanced Malware	**x**		
	Firmware Hacks	**x**		
	Device Tampering	**x**		
	Spyware	**x**		
	IoT Botnets	**x**		
	Ransomware	**x**		
	Battery Draining Attack	**x**		
	Identification Attack	**x**		
	Privacy Breach	**x**		

## Footnotes

1 5G-Xhaul project weblink: https://www.5g-xhaul-project.eu/
